# Experiences of violence among adolescent girls and young women in Nairobi’s informal settlements prior to scale-up of the DREAMS Partnership: Prevalence, severity and predictors

**DOI:** 10.1371/journal.pone.0231737

**Published:** 2020-04-22

**Authors:** Benedict O. Orindi, Beatrice W. Maina, Sheru W. Muuo, Isolde Birdthistle, Daniel J. Carter, Sian Floyd, Abdhalah Ziraba

**Affiliations:** 1 Kenya Medical Research Institute–Wellcome Trust Research Programme, Kilifi, Nairobi, Kenya; 2 African Population and Health Research Center, Nairobi, Kenya; 3 Department of Public Health and Primary Care, Leuven Biostatistics and Statistical Bioinformatics Centre, Katholieke Universiteit Leuven, Leuven, Belgium; 4 Global Programs for Research and Training, University of California, San Francisco (UCSF), Nairobi, Kenya; 5 London School of Hygiene and Tropical Medicine, London, United Kingdom; Centre for Sexual Health & HIV/AIDS Research, ZIMBABWE

## Abstract

**Introduction:**

We sought to estimate the prevalence, severity and identify predictors of violence among adolescent girls and young women (AGYW) in informal settlement areas of Nairobi, Kenya, selected for DREAMS (Determined Resilient Empowered AIDS-free, Mentored and Safe) investment.

**Methods:**

Data were collected from 1687 AGYW aged 10–14 years (n = 606) and 15–22 years (n = 1081), randomly selected from a general population census in Korogocho and Viwandani in 2017, as part of an impact evaluation of the “DREAMS” Partnership. For 10–14 year-olds, we measured violence experienced either in the past 6 months or ever using a different set of questions from those used for 15–22 year-olds. Among 15–22 year-olds we measured prevalence of violence, experienced in the past 12 months, using World Health Organization (WHO) definitions for violence typologies. Predictors of violence were identified using multivariable logit models.

**Results:**

Among 606 girls aged 10–14 years, about 54% and 7% ever experienced psychological and sexual violence, respectively. About 33%, 16% and 5% experienced psychological, physical and sexual violence in the past 6 months. The 10–14 year old girls who engaged in chores or activities for payment in the past 6 months, or whose family did not have enough food due to lack of money were at a greater risk for violence. Invitation to DREAMS and being a non-Christian were protective. Among 1081 AGYW aged 15–22 years, psychological violence was the most prevalent in the past year (33.1%), followed by physical violence (22.9%), and sexual violence (15.8%). About 7% experienced all three types of violence. Severe physical violence was more prevalent (13.8%) than moderate physical violence (9.2%). Among AGYW aged 15–22 years, being previously married/lived with partner, engaging in employment last month, food insecure were all risk factors for psychological violence. For physical violence, living in Viwandani and being a Muslim were protective; while being previously married or lived with a partner, or sleeping hungry at night during the past 4 weeks were risk factors. The odds of sexual violence were lower among AGYW aged 18–22 years and among Muslims. Engaging in sex and food insecurity increased chances for sexual violence.

**Conclusions:**

Prevalence of recent violence among AGYW is high in this population. This calls for increased effort geared towards addressing drivers of violence as an early entry point of HIV prevention effort in this vulnerable group.

## Introduction

Violence against adolescent girls and young women (AGYW) is a global phenomenon and has implications on their wellbeing. According to the World Health Organization (1) more than a third of women globally have experienced either physical or sexual violence or both from an intimate partner or non-partner in their lifetime. It has also been reported that about one in three ever-partnered AGYW aged 15–24 years have experienced physical and/or sexual violence by an intimate partner [1]. In sub-Saharan Africa, about 37 percent of ever-partnered women have experienced physical and/or sexual violence [1].

A report by the United Nations Children’s Fund Kenya County Office indicated that in 2010 about 76 percent of AGYW aged between 18 and 24 years had experienced sexual, physical or emotional violence prior to age 18 years while about half of AGYW aged 13 to 17 years had experienced sexual or physical violence in the 12 months preceding the study [2]. Exposure to violence has deleterious effects on AGYWs’ health outcomes, including their mental, sexual and reproductive as well as social and economic wellbeing. It is one of the leading causes of death in this age group globally [3–6]. Kabiru and colleagues found that adolescent girls aged 11–15 years who had experienced violence had lower expectations of achieving their aspirations compared to those who had not experienced violence [5]. A study conducted in Malawi and South Africa showed that exposure to violence has an impact on school enrolment and performance [7].

Studies looking at risk factors for violence have found that women’s economic status, and norms that place women’s status in society at a lower position have a significant association with violence experience [4, 8]. In their review of risk factors for AGYW’s experience of violence in romantic relationships, Vezina and Hebert (4) also found that adolescent mothers and those who had dropped out of school were at a greater risk of experiencing dating violence. Having witnessed violence has been found to be a risk factor for violence experience [4, 9, 10]. Vezina and Hebert (4) argue that having witnessed violence as a child may contribute to perceiving violence as an acceptable way of resolving conflict. This is in line with the Social Learning Theory which postulates that individuals model behavior based on what they observe from their environments/contexts [11].

Living in urban informal settlements has been found to heighten risk of experiencing violence. Kabiru, Mumah (5) found that about one in three girls aged 11–15 years in urban informal settlements of Nairobi has experienced at least one form of gender-based violence. Urban informal settlements have been associated with high levels of poverty, crime and violence, poor health outcomes—including HIV/AIDS—as well as poor access to basic services such as schools and health care facilities. Past studies have found strong association between violence experience and poverty at the household [9, 12] and community levels [9, 13, 14], a major characteristic of the urban informal settlements.

Exposure to violence has been found to be associated with HIV acquisition [15], while HIV-positive status is thought to provoke violence in some contexts [8, 16, 17]. In their review, Campbell et al. argue that increased risk for HIV acquisition works through increased sexual risk-taking behaviors, forced sex with an infected partner, and compromised negotiation on safe sex practices [18]. AGYW who have been exposed to violence may have low negotiation powers, thus compromised safe sex practices.

In the slum context, the risk of HIV and occurrence of interpersonal violence risk seem to be heightened. While recent data are sparse, a study conducted in 2007 showed that the HIV prevalence was higher at about 12% among slum residents compared to 5% and 6% among non-slum urban and rural residents, respectively, and this mirrored the national trend where the burden was higher among females [19]. Impersonal violence including violence against women is common slums compared to the general Kenyan population [20].

The HIV epidemic in sub-Saharan Africa cannot be brought under control without reducing HIV acquisition among AGYW, the most rapidly expanding demographic group on the continent [8]. The DREAMS Partnership is an initiative aimed at reducing the incidence of HIV among AGYW in 10 sub-Saharan African countries. It supports a core package of interventions provided “at scale” targeting AGYW, their families, wider communities, including men who are the sexual partners of AGYW [21]. The core package includes interventions aimed at addressing HIV risk behaviors, HIV transmission, socio-economic vulnerabilities and gender-based violence. The interventions are aimed at empowering girls and young women to reduce their risk of HIV infection, and include SASA! (Start, Awareness, Support, Action) and school-based HIV and violence prevention programs. The core package interventions are described in detail elsewhere [21].

Nairobi, Kenya, is among the 4 sites chosen for an independent impact evaluation [22]. Using enrolment interview data from AGYW cohorts established for the impact evaluation, this analysis aims to summarize the prevalence of experience of violence, the severity of physical violence, and to identify factors associated with experience of violence among AGYW living in the Korogocho and Viwandani informal settlements of Nairobi, within the first year of DREAMS implementation. We also sought to determine whether DREAMS is reaching those who experience violence, by comparing those who were and were not invited to participate in DREAMS.

## Methods

### Study design, setting and sample

The DREAMS impact evaluation design and data collection protocol have been described in detail elsewhere [22]. Briefly, the design leverages the Nairobi Urban Health and Demographic Surveillance System (NUHDSS), a longitudinal platform run by the African Population and Health Research Center (APHRC) in two informal settlements of Korogocho and Viwandani since 2002 [23]. Both informal settlements are characterized by high levels of unemployment, sub-standard housing and crowding, limited access to education and other social services, high levels of insecurity, and inadequate water and sanitation infrastructure. Whereas Korogocho is a more settled community with many long term residents, the population in Viwandani is more mobile and youthful [23]. In spite of this known fact we took measures (such as making multiple visits, altering time of visits, and re-assigning more experienced field staff) to reach as many sample participants as possible.

We used different interview tools for the age groups 10–14 years and 15–22 years, as DREAMS had a different primary package for each age group, but also some of the questions in the tool for the former group could not be asked to girls in the latter group as they were inappropriate given their age. The tool for the 10–14 years was adopted from the Global Early Adolescent Study (GEAS) tool, and has been validated for this particular age group in our setting. For the 15–22 years, the World Health Organization violence against women (WHO VAW) instrument [24] was included to measure exposure to violence. In this paper, we used round one (enrolment) data collected March–July 2017 on a randomly selected sample of AGYW aged 10–22 years. For the 10–14 year-olds, a random list of 1017 girls was generated. Of these, 333 (32.7%) were no longer eligible at the time of visit. Of the remaining 684, 46 (6.7%) were absent for extended period of time, 9 (1.3%) either refused or their parents refused their participation, and 23 (3.4%) had their structures located but respondents' whereabouts were unknown, leaving 606 (88.6%) with successful interviews. At enrolment, we targeted a minimum sample of 1000 AGYW aged 15–22 years. A random list of 2599 AGYW was generated. Of these, 695 (26.7%) were no longer eligible at the time of visit. Of the remaining 1904, 6 (0.31%) had incomplete interviews, 315 (16.5%) were absent from their residence for extended period of time, 283 (14.9%) refused to participate either by self or their parents/guardians, and 219 (11.5%) had their structures located but respondents' whereabouts were unknown, leaving 1081 (56.8%) with successful interviews. Our assessment of how those who participated compared to those who did not showed that there were no major differences by several socio-demographic characteristics indicating that there was unlikely to have been selection bias.

### Measures

### Outcome variables

Among girls aged 10–14 years, experience of violence was measured using the questions listed in Box 1. The questions were of two types: 1) Whether the girl has “ever experienced …”; and 2) whether the girl “experienced in the last six months …”. They were classified into psychological violence (questions 1, 2, and 6), sexual violence (questions 3, 4 and 5), and physical violence (question 7). A girl was considered to have experienced violence if she gave any of the answers “sometimes” (= 1), “often” (= 2) for questions 1–4; or “yes” (= 1) for question 5; or “yes, by both boy and girl” (= 1), “yes, by boy” (= 2), or “yes, by girl” (= 3) for questions 6 and 7. As these questions were not similar as those used for AGYW aged 15–22 years, the data for this group were analyzed separately. Consequently, the results for the two age categories are not immediately comparable.

**Box 1. The items used to measure experience of violence among the early adolescent girls 10–14 years old**

**Table:** 

Items*
1. Have you ever been scared or felt really bad because grown-ups called you names, said mean things to you, or said they didn’t want you? [Psychological violence]
2. Have you ever been scared that your parents or other adults were going to hurt you badly (so that you were injured or killed)? [Psychological violence]
3. Has an adult ever touched you in your private parts except when being bathed? [Sexual violence]
4. Has an adult ever attempted or forced you to have sexual intercourse? [Sexual violence]
5. During the last six months, has someone touched you in a way that you did not want to be touched? [Sexual violence]
6. During the last six months, have you been teased or called names by someone? [Psychological violence]
7. During the last 6 months have you ever been slapped, hit or otherwise been physically hurt by a boy or girl in a way that you did not want? [Physical violence]

*Questions 1–4 had the answer categories “Never” (= 0), “Sometimes” (= 1), “Often” (= 2), “Don’t know” (= 999), “Refused” (= 996); Question 5 had the answers “No” (= 0), “Yes” (= 1), “Can’t remember” (= 998), “Don’t know” (= 999), “Refused to answer” (= 996); Questions 6 and 7 had answer options of “No” (= 0), “Yes, by both boys and girls” (= 1), “Yes, by boys” (= 2), “Yes, by girl or girls” (= 3), “Cannot remember” (= 998), “Don’t know” (= 999), “Refused” (= 996).

Among the AGYW aged 15–22 years, experience of violence was measured using 15 questions on a binary scale of “yes” (= 1) or “no” (= 0). The questions read like: “Has any male ever done any of the following things to you in the past 12 months?” (see Box 2). The tool has shown good psychometric properties [24–29]. The WHO classified these items into three dimensions of violence [24]: psychological violence (items 1 to 3; Cronbach’s alpha = 0.67), physical violence (items 4 to 11; Cronbach’s alpha = 0.77), and sexual violence (items 12 to 15; Cronbach’s alpha = 0.79).

**Box 2. The 15 items used to measure experience of violence in the past 12 months among AGYW aged 15–22 years and the hypothesized domains**

**Table:** 

Items*
**Psychological (Emotional) violence**
1. Say or do something to humiliate you in front of others?
2. Threaten to hurt or harm you or someone close to you?
3. Insult you or make you feel bad about yourself?
**Physical violence**
4. Push you, shake you, or throw something at you?
5. Slap you?
6. Twist your arm or pull your hair?
7. Punch you with his fist or something that could hurt you?
8. Kick you, drag you, or beat you up?
9. Try to choke you or burn you on purpose?
10. Threatened to attack you with a knife or other weapon?
11. Attacked you with a weapon?
**Sexual violence**
12. Touched you in a sexual way (e.g. kissing, grabbing, or fondling), when you did not want them to?
13. Try to have sexual intercourse with you when you did not want to but did not succeed?
14. Physically forced you to have sexual intercourse even when you did not want to?
15. Forced you to perform sexual acts when you did not want to?

*The answer options were “yes” (= 1) or “no” (= 0)

### Explanatory variables

Explanatory variables included self-reported invitation to participate in DREAMS, slum of residence (site), age at survey, marital status, whether the girl is currently in school, educational level, religion, ethnicity (i.e., mainly Somali, Kisii, Kamba, Kikuyu, Luhya, and Luo), recent employment/engagement in income generating activity, ever had sex, ever been pregnant, ever given birth, slept hungry at night in past 4 weeks, self-assessed household economic situation, and wealth index. For ethnicity, categories with low frequencies were grouped under “other. Wealth index was constructed using principle component analysis (PCA) with input as indicator variables on ownership of household and individual assets/items (including ownership of television, electricity, fridge, radio, bicycle, motorcycle, shoes, blanket, clothes, among others), household structure (i.e., floor, roof, and wall material), and on household’s water supply and sanitation [30, 31]. The wealth index was split into three categories of “poor” (= 1), “medium” (= 2), and “wealthy” (= 3). The variables ‘ever been pregnant’ and ‘ever given birth’ were excluded from the present analysis because of multicollinearity (see S1 Table). Data on the gender of the teacher were not collected for the 15–22 year-olds.

Data were collected electronically using face-to-face interviews by well trained and experienced field interviewers and supervisors who were also well conversant with the study area. For the 10–14 years cohort, only selected female interviewers surveyed them to minimize potential response bias. For the 15–24 year-old cohort, enumerators were both males and females with mean age 27 years and 24 years, respectively. The mix of gender among the interviewers catered for any respondent that may have preferred to be interviewed by a particular gender. Prior to the survey, tools were translated into Kiswahili only, and were back translated to ensure the questions did not lose their meaning; the tools were piloted and issues that arose were addressed.

### Analysis

To estimate the prevalence of violence, we obtained, for each domain, the proportion of AGYW who reported to have experienced at least one of the acts of violence comprising that domain. These proportions were summarized with respect to important demographic characteristics. We also compared those who were and were not invited to participate in DREAMS.

To assess the severity of violence, a woman/girl was considered to have experienced “moderate” violence if she answered “yes” to one or more of questions 4–5 in Box 2 (and does not answer “yes” to questions 6–11). A woman/girl was considered to have experienced “severe” violence if she answered “yes” to one or more of questions 6 to 11 [24]. We note here that this could only be done for physical violence (and among the 15–22 year olds only) as WHO guidelines on violence classification as moderate or severe only exist for physical violence, to our best knowledge [24, 25]. However, in another study [1], all acts of sexual violence were considered severe but it is not clear what items comprised sexual violence domain. We return to this in the Discussion section.

To investigate factors associated with violence, we considered psychological and sexual violence outcomes as dichotomous, that is, “not abused” (= 0) or “abused” (= 1). Physical violence was considered ordinal with three levels of “none” (= 0), “moderate” (= 1), and “severe” (= 2), as described above. Ordinal regression analysis was used [32]. For each of the three violence outcomes, we evaluated three plausible link functions, that is, logit, probit, and complementary log-log (clog-log). Based on the log-likelihood values and practical considerations, the logit link was chosen (see Summary of model fit evaluation in Supporting Information, S1 Text). Thus, all inferences presented in the next section were based on models with a logit link. We adopted a three-step approach to the analyses. First, a model was run with one explanatory variable at a time (‘model 1’). Next, a model was fitted for explanatory variable adjusted for Invitation to DREAMS, site, and age (‘model 2’). Finally, all explanatory variables significant at p≤0.10 in the second step (i.e., in model 2) were included in a multivariable model (model 3). Explanatory variables were tested in the multivariable model using likelihood ratio test (LRT) at p≤0.05 significance level. Invitation to DREAMS, site, and age were retained in the multivariable model even if they were not significant, as we wished to adjust for their effect. Data management and all analyses were performed using STATA v14 (StataCorp, College Station, TX). Computational details and model fit evaluation are presented in Supporting Information, S1 Text.

### Ethical considerations

Ethics approval was provided by the London School of Hygiene & Tropical Medicine (LSHTM; Ref 211 11835) and AMREF Health Africa (No ESRC P298/2016). Written informed consent was obtained from each participant. For legal minors, assent was obtained from the minor before the guardian gave consent. The participants were given copies of the consent documents and project information sheet. The interviewer took them through the information sheet and consent form and gave them an opportunity to ask questions. Once they indicated to have understood about the project and agreed to participate, they were requested to sign the certificate of consent. Incase the respondents were not able to write they had their thumb print on the consent form (round 1), or put a mark on the soft copy of the consent (rounds 2 and 3) and the field interviewer noted on the comments section that the respondent was unable to write.

## Results

### Demographics of the adolescent girls and young women

Data were available on 606 (10–14years) and 1081 (15–22 years) AGYW. The mean (median) age for the 10–14 year olds was 12.1 years (12 years), and that of the 15–22 year olds was 17.9 years (17.0 years). Of 606 girls aged 10–14 years, 53% (n = 323) were from Korogocho and 47% (n = 283) were from Viwandani. Five of the girls were not enrolled in school (~1%). The majority were Christians (88.1%, n = 534), 10% were Muslims. They were of different ethnic origins including Kikuyu (32.2%, n = 195), Luo (19%, n = 115), Kamba (15.8%, n = 96), Luhya (15%, n = 91), Somali (9.2%, n = 56), and Kisii (5.6%, n = 34). About 5% (n = 29) had done chores or activities for which they got paid money over the past 6 months (e.g., worked for neighbors or friends, day labor or temporary work, worked for family (such as parents or relatives), providing services, among others).

Of 1081 AGYW aged 15–22 years, about half (n = 536) reported to have ever been invited to participate in DREAMS. The majority had never been married (78%; 843/1081), were in school (57.8%; 625/1081), were Christians (84.8%; 917/1081), had never engaged in an income generating activity (71.8%; 776/1081), had had sex (59.4%; 642/1081), and assessed their household economic situation as moderately poor. The majority of AGYW were from the Kikuyu (29.5%, n = 319) and Kamba (19.2%, n = 208) communities, followed by Luos (16.3%, n = 176) and Luhyas (16.3%, n = 176), Somalis (8.3%, n = 90), and Kisiis (4.7%, n = 51). About nine in ten AGYW reported to be knowing their HIV statuses.

### Prevalence among 10–14 year olds

Table 1 shows, for girls aged 10–14 years, the proportions that experienced violence in the past 6 months or ever experienced violence by demographic characteristics. Overall, psychological violence was the most prevalent, both within the past 6 months (32.8%) and life time (54%), followed by physical violence (16.3%), and sexual (7.1%). Violence was generally less prevalent among those invited to DREAMS, currently enrolled in school, did not engage in chores or activities for payment during the past 6 months, had never had sex, or family had enough food. S2 Table shows that the proportions that experienced any form of violence in the past 6 months was 37.8%, while in life time was 57.4%.

**Table 1:** Percent of girls aged 10–14 years reporting to have experienced violence in past 6 months or ever experienced violence.

		Experienced violence past 6 months						Ever experienced			
	Total	Psychological		Physical*		Sexual		Psychological		Sexual	
Characteristics	N	n	%	n	%	n	%	n	%	n	%
Overall	606	199	32.8	99	16.3	28	4.6	327	54.0	43	7.1
Invitation to DREAMS											
Not invited	316	107	33.9	53	16.8	16	5.1	183	57.9	26	8.2
Invited	290	92	31.7	46	15.9	12	4.1	144	49.7	17	5.9
DSS study site											
Korogocho	323	110	34.1	50	15.5	18	5.6	163	50.5	25	7.7
Viwandani	283	89	31.4	49	17.3	10	3.5	164	58.0	18	6.4
Age groups											
10–12 years	372	123	33.1	64	17.2	22	5.9	208	55.9	30	8.1
13–14 years	234	76	32.5	35	15.0	6	2.6	119	50.9	13	5.6
Currently enrolled in school?											
No	5	2	40.0	2	40.0	1	20.0	3	60.0	1	20.0
Yes	601	197	32.8	97	16.1	27	4.5	324	53.9	42	7.0
School grade											
Upper primary or Secondary	257	80	31.1	33	12.8	8	3.1	131	51.0	14	5.4
Middle primary	311	108	34.7	58	18.6	16	5.1	177	56.9	24	7.7
Lower primary	38	11	28.9	8	21.1	4	10.5	19	50.0	5	13.2
School type											
Public school	271	94	34.7	49	18.1	7	2.6	153	56.5	14	5.2
Private, non-religious or secular school	260	75	28.8	36	13.8	17	6.5	130	50.0	25	9.6
Religious school	70	28	40.0	12	17.1	3	4.3	41	58.6	3	4.3
Not enrolled in school	5	3	60.0	2	40.0	1	20.0	2	40.0	1	20.0
Gender of teachers											
Mostly women (very few or no men)	162	44	27.2	23	14.2	7	4.3	78	48.1	12	7.4
Mostly men (very few or no women)	58	17	29.3	11	19.0	3	5.2	28	48.3	4	6.9
Both men and women	381	136	35.7	63	16.5	17	4.5	218	57.2	26	6.8
Religion											
Christian	534	180	33.7	93	17.4	27	5.1	298	55.8	40	7.5
Muslim	61	16	26.2	4	6.6	1	1.6	24	39.3	3	4.9
Other	11	3	27.3	2	18.2	0	0.0	5	45.5	0	0.0
Ethnic group											
Somali	56	15	26.8	4	7.1	1	1.8	22	39.3	4	7.1
Kamba	96	33	34.4	15	15.6	3	3.1	52	54.2	4	4.2
Kikuyu	195	59	30.3	34	17.4	10	5.1	100	51.3	12	6.2
Kisii	34	10	29.4	7	20.6	3	8.8	24	70.6	5	14.7
Luhya	91	32	35.2	18	19.8	3	3.3	56	61.5	6	6.6
Luo	115	44	38.3	19	16.5	8	7.0	62	53.9	12	10.4
Other	19	6	31.6	2	10.5	0	0.0	11	57.9	0	0.0
Activities for payment past 6 months											
no	577	183	31.7	89	15.4	22	3.8	305	52.9	34	5.9
yes	29	16	55.2	10	34.5	6	20.7	22	75.9	9	31.0
Ever had sex											
no	594	192	32.3	93	15.7	22	3.7	316	53.2	35	5.9
yes	12	7	58.3	6	50.0	6	50.0	11	91.7	8	66.7
Family did not have enough food due to money											
no	228	66	28.9	28	12.3	8	3.5	114	50.0	11	4.8
yes	378	133	35.2	71	18.8	20	5.3	213	56.3	32	8.5

*Ever experienced and experienced violence in past 6 months are the same for physical violence as it was based on a single item measuring experience of that act in past 6 months.

### Predictors of violence among 10–14 year-olds

S4 Table summarizes predictors for violence among girls of ages 10–14 years. After adjusting for other factors, the odds of experiencing any violence were greater among girls who engaged in chores or activities for payment in the past 6 months, and among those whose family did not have enough food due to lack of money. Violence was lower among girls invited to DREAMS, and among non-Christians.

### Prevalence of violence among the 15–22 year-olds

**Fig 1:**
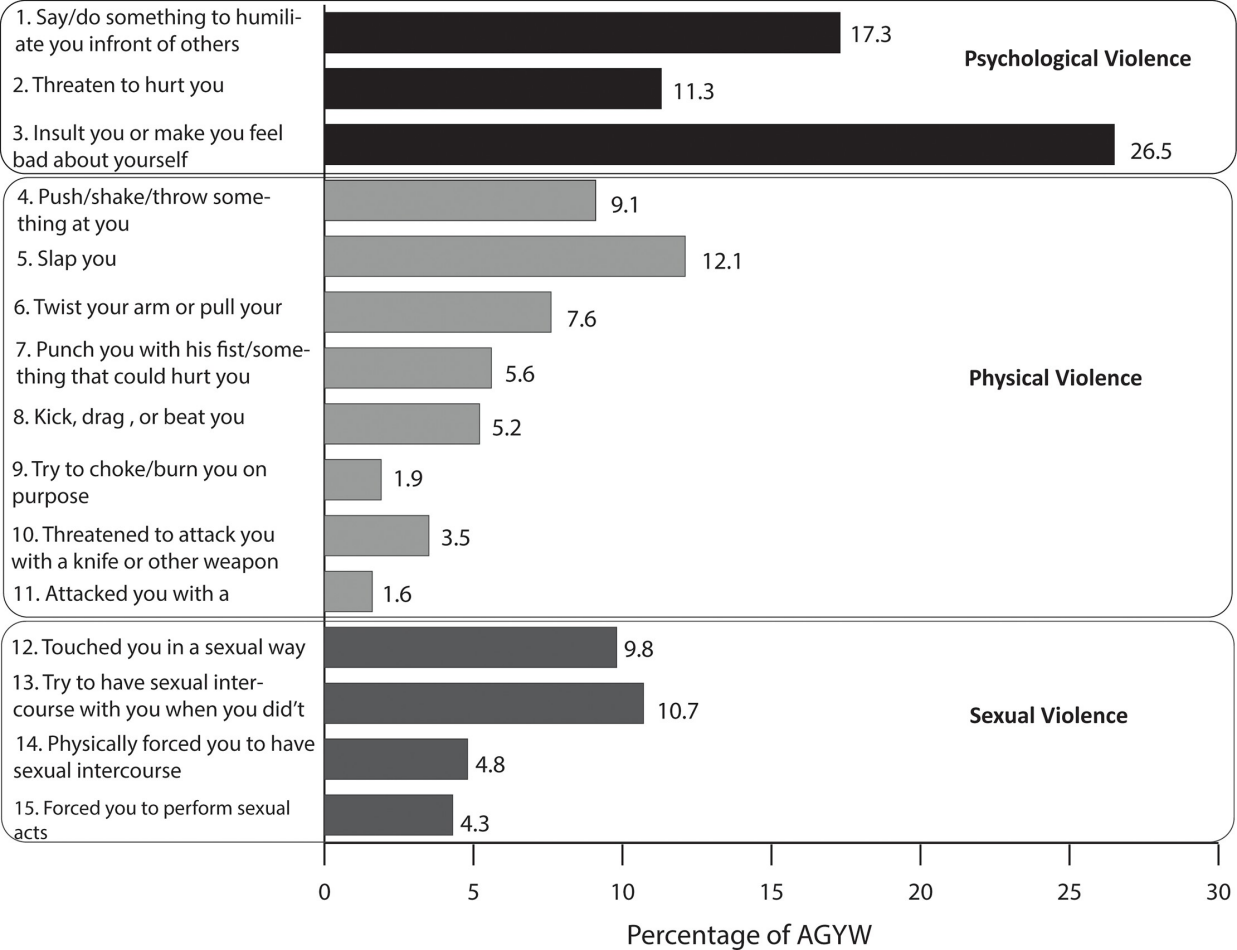
Percent of AGYW (15–22 years) who experienced violence in the past 12 months.

Fig 1 shows the percentage of the 15–22 year-old cohorts with a “yes” answer to each of the 15 violence questions listed in Box 1. Item 3 (“insult you or make you feel bad about yourself”) was the most experienced at 26.5%; followed by item 1 (“Say or do something to humiliate you in front of others”, 17.3%); then items 2 (“Threaten to hurt or harm you or someone close to you”), 5 (“Push you, shake you, or throw something at you”), 12 (“Touched you in a sexual way (e.g. kissing, grabbing, or fondling), when you did not want them to”), and 13 (“Try to have sexual intercourse with you when you did not want to but did not succeed”) at about 10%.

**Fig 2:**
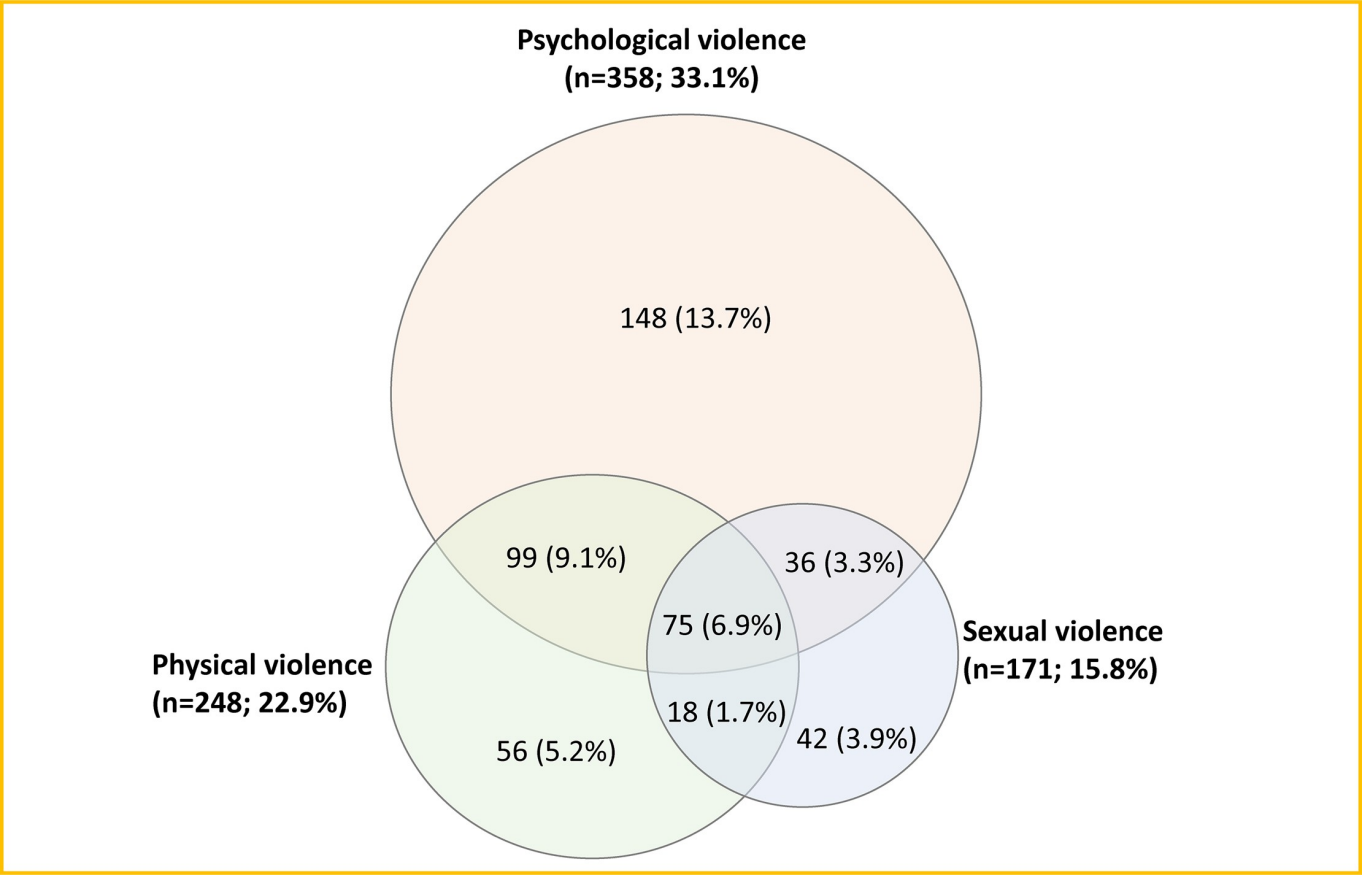
Number of AGYW (15–22 years) who experienced psychological, physical, and sexual violence and more than one type of violence during the past 1 year.

Fig 2 shows that the prevalence of psychological violence among at the 15–22 year-olds was 33.1% (95%CI 30.4–36.0%; n = 358), followed by physical violence (22.9%, 95%CI 20.5–25.5%; n = 248), and sexual violence (15.8%, 95%CI 13.8–18.1%; n = 171), in the past 12 months. About 44% experienced at least one of the 15 acts of violence in the past 12 months. Further, some of the AGYW experienced more than one type of violence, with 6.9% (95%CI 5.56–8.62%, n = 75) experiencing all three types of violence. About 9.2% (n = 99) reported both physical and psychological, 3.33% (n = 36) reported both psychological and sexual, and 1.7% (n = 18) reported both physical and sexual violence. Tables 2–4 present the prevalence of the three violence domains by demographic characteristics. They show that within each violence measure the prevalence of violence varied across the demographic characteristics, with about 45% and 33% of those who, respectively, know and don’t know own HIV status experiencing any form of violence.

**Table 2:** Prevalence and predictors of psychological violence among 15–22 years AGYW.

	Total	Experienced Psychological violence n (%)	Model 1	Model 3	
Explanatory variable	N		Unadjusted OR (95%CI)	Fully adjusted OR (95%CI)	p-value
Overall	1081	358 (33.1)			
Invited to DREAMS			P = 0.652	P = 0.493	
Not invited	545	177 (32.5)	1	1	
Invited	536	181 (33.8)	1.06 (0.82–1.37)	1.1 (0.83–1.46)	0.493
Site/slum			P = 0.760	P = 0.882	
Korogocho	617	202 (32.7)	1	1	
Viwandani	464	156 (33.6)	1.04 (0.81–1.34)	1.02 (0.75–1.4)	0.882
Age (years)			P = 0.292	P = 0.764	
15–17	547	174 (31.8)	1	1	
18–22	534	184 (34.5)	1.15 (0.89–1.48)	0.95 (0.67–1.34)	0.764
Marital/co-habitation status			P = 0.001	P = 0.002	
Never married	843	278 (33.0)	1	1	
Previously married/lived with partner	33	21 (63.6)	3.58 (1.73–7.37)	2.35 (1.06–5.21)	0.035
Currently married/living with partner	205	59 (28.8)	0.85 (0.61–1.18)	0.62 (0.40–0.96)	0.030
Currently in school			P = 0.080		
No	455	158 (34.7)	1		
Yes	626	200 (31.9)	0.87 (0.67–1.12)		
Educational level			P = 0.335		
None/some primary	124	36 (29.0)	1		
Complete primary	217	62 (28.6)	0.95 (0.59–1.54)		
Some secondary	491	175 (35.6)	1.31 (0.85–2.00)		
Complete secondary	198	70 (35.4)	1.30 (0.80–2.11)		
Tertiary: university/college/vocational	51	15 (29.4)	1.02 (0.50–2.09)		
Religion			P<0.001	P = 0.163	
Christian	917	333 (36.3)	1	1	
Muslim	142	16 (11.3)	4.49 (2.62–7.68)	0.35 (0.12–1.03)	0.057
Other	22	9 (40.9)	5.45 (2.01–14.77)	0.99 (0.4–2.44)	0.976
Ethnicity			P<0.001	P = 0.724	
Somali	90	9 (10)	1	1	
Kamba	208	71 (34.1)	4.76 (2.26–10.04)	1.73 (0.47–6.41)	0.411
Kikuyu	319	109 (34.2)	4.67 (2.26–9.66)	1.57 (0.44–5.66)	0.488
Kisii	51	21 (41.2)	6.3 (2.6–15.28)	2.31 (0.56–9.47)	0.245
Luhya	176	62 (35.2)	4.89 (2.3–10.41)	1.56 (0.42–5.7)	0.505
Luo	176	74 (42)	6.38 (3.01–13.52)	2.03 (0.55–7.46)	0.288
Other	61	12 (19.7)	2.20 (0.87–5.61)	1.46 (0.51–4.19)	0.481
Ever & recent employment/income generating activity			P<0.001	P = 0.028	
Never	776	235 (30.3)	1	1	
Yes, not in last month	172	58 (33.7)	1.17 (0.82–1.66)	1.03 (0.68–1.56)	0.878
Yes, in last month	133	65 (48.9)	2.20 (1.52–3.20)	1.76 (1.14–2.7)	0.010
Ever had sex			P<0.001	P = 0.140	
No	439	192 (43.7)	1	1	
Yes	642	166 (38.0)	1.47 (1.13–1.9)	1.34 (0.91–1.99)	0.140
Slept hungry at night past 4 weeks			P = 0.002	P = 0.016	
No	729	223 (30.6)	1	1	
Yes	352	135 (38.4)	1.40 (1.08–1.83)	1.44 (1.07–1.94)	0.016
Self-assessed household economic situation			P = 0.907		
Very poor	139	46 (33.1)	1		
Moderately poor	858	286 (33.3)	1.01 (0.69–1.48)		
Not poor	84	26 (31.0)	0.91 (0.51–1.62)		
Wealth quantile			P = 0.790		
Poor	361	123 (34.1)	1		
Medium	360	115 (31.9)	0.90 (0.66–1.22)		
Wealthy	360	120 (33.3)	0.94 (0.69–1.29)		
Knows HIV status					
No	133	31 (33.3)	1		
Yes	948	327 (34.5)	1.73 (1.13–2.65)		

Model 1 is unadjusted logit model; Model 2 is invited to DREAMS, site, and age adjusted logit model for each predictor; Model 3 is fully adjusted multivariable logit model. Variables significant at P<0.10 in model 2 were included in model 3. Invited to DREAMS, site, and age were included even if they were not significant as we wished to adjust for their impact. The capital P is likelihood ratio rest (LRT) p-value

**Table 3:** Prevalence and predictors of physical violence among 15–22 years AGYW.

	Total	Experienced physical violence n (%)	Model 1	Model 3	
Explanatory variable	N		Unadjusted OR (95%CI)	Fully adjusted OR (95%CI)	p-value
Overall	1081	248 (22.9)			
Invited to DREAMS			P = 0.792	P = 0.612	
Not invited	545	124 (22.8)	1	1	
Invited	536	124 (23.1)	1.04 (0.78–1.38)	1.08 (0.8–1.47)	0.612
Site/slum			P<0.001	P<0.001	
Korogocho	617	172 (27.9)	1	1	
Viwandani	464	76 (16.4)	0.50 (0.37–0.68)	0.46 (0.34–0.64)	<0.001
Age (years)			P = 0.494	P = 0.068	
15–17	547	119 (21.8)	1	1	
18–22	534	129 (24.2)	1.10 (0.83–1.46)	0.70 (0.47–1.03)	0.068
Marital/co-habitation status			P = 0.002	P = 0.109	
Never married	843	178 (21.1)	1	1	
Previously married/lived with partner	33	15 (45.5)	3.23 (1.63–6.41)	2.24 (1.05–4.76)	0.037
Currently married/living with partner	205	55 (26.8)	1.31 (0.93–1.86)	1.25 (0.78–2.00)	0.345
Currently in school			P = 0.010	P = 0.612	
No	455	122 (26.8)	1	1	
Yes	626	126 (20.1)	0.69 (0.52–0.92)	0.9 (0.6–1.36)	0.612
Educational level			P = 0.118		
None/some primary	124	35 (28.2)	1		
Complete primary	217	56 (25.8)	0.87 (0.54–1.42)		
Some secondary	491	108 (22)	0.70 (0.45–1.08)		
Complete secondary	198	41 (20.7)	0.61 (0.37–1.02)		
Tertiary: university/college/vocational	51	8 (15.7)	0.4 (0.16–0.97)		
Religion			P = 0.016	P = 0.005	
Christian	917	223 (24.3)	1	1	
Muslim	142	19 (13.4)	2.08 (1.25–3.44)	0.41 (0.24–0.7)	0.001
Other	22	6 (27.3)	2.47 (0.87–7.05)	0.88 (0.33–2.33)	0.792
Ethnicity			P = 0.007		
Somali	90	12 (13.3)	1		
Kamba	208	40 (19.2)	1.53 (0.76–3.07)		
Kikuyu	319	73 (22.9)	1.94 (1–3.74)		
Kisii	51	12 (23.5)	1.96 (0.81–4.74)		
Luhya	176	43 (24.4)	2.14 (1.07–4.29)		
Luo	176	57 (32.4)	3.26 (1.65–6.44)		
Other	61	11 (18.0)	1.49 (0.61–3.63)		
Ever & recent employment/income generating activity			P = 0.139		
Never	776	166 (21.4)	1		
Yes, not in last month	172	43 (25.0)	1.20 (0.82–1.75)		
Yes, in last month	133	39 (29.3)	1.48 (0.99–2.22)		
Ever had sex			P = 0.001	P = 0.058	
No	439	124 (19.3)	1	1	
Yes	642	124 (28.4)	1.63 (1.23–2.17)	1.56 (0.98–2.47)	0.058
Slept hungry at night past 4 weeks			P = 0.001	P = 0.043	
No	729	144 (19.8)	1	1	
Yes	352	104 (29.5)	1.67 (1.25–2.23)	1.38 (1.01–1.89)	0.043
Self-assessed household economic situation			P = 0.311		
Very poor	139	26 (18.7)	1		
Moderately poor	858	199 (23.2)	1.29 (0.82–2.02)		
Not poor	84	23 (27.4)	1.64 (0.87–3.09)		
Wealth quantile			P<0.001		
Poor	361	109 (30.2)	1		
Medium	360	72 (20.0)	0.57 (0.41–0.8)		
Wealthy	360	67 (18.6)	0.55 (0.39–0.78)		
Knows HIV status					
No	133	24 (18.0)	1		
Yes	948	224 (23.6)	1.41 (0.88–2.24)		

Model 1 is unadjusted logit model; Model 2 is invited to DREAMS, site, and age adjusted logit model for each predictor; Model 3 is fully adjusted multivariable logit model. Variables significant at P<0.10 model 2 were included in model 3. Invited to DREAMS, site, and age were included even if they were not significant as we wished to adjust for their impact. The capital P is likelihood ratio rest (LRT) p-value.

**Table 4:** Prevalence and predictors of sexual violence among 15–22 years AGYW.

	Total	Experienced Sexual violence n (%)	Model 1	Model 3	
Explanatory variable	N		Unadjusted OR (95%CI)	Fully adjusted OR (95%CI)	p-value
Overall	1081	171 (15.8)			
Invited to DREAMS			P = 0.473	P = 0.593	
Not invited	545	90 (16.5)	1	1	
Invited	536	81 (15.1)	0.89 (0.64–1.23)	0.90 (0.62–1.32)	0.593
Site/slum			P = 0.996	P = 0.845	
Korogocho	617	98 (15.9)	1	1	
Viwandani	464	73 (15.7)	1 (0.72–1.39)	1.04 (0.70–1.55)	0.845
Age (years)			P = 0.241	P = 0.028	
15–17	547	79 (14.4)	1	1	
18–22	534	92 (17.2)	1.22 (0.88–1.69)	0.59 (0.36–0.94)	0.028
Marital/co-habitation status			P = 0.762		
Never married	843	130 (15.4)	1		
Previously married/lived with partner	33	6 (18.2)	1.23 (0.5–3.04)		
Currently married/living with partner	205	35 (17.1)	1.14 (0.76–1.72)		
Currently in school			P = 0.009	P = 0.160	
No	455	88 (19.3)	1	1	
Yes	626	83 (13.3)	0.65 (0.47–0.9)	1.41 (0.87–2.28)	0.160
Educational level			P = 0.247		
None/some primary	124	19 (15.3)	1		
Complete primary	217	30 (13.8)	0.84 (0.46–1.56)		
Some secondary	491	72 (14.7)	0.90 (0.53–1.55)		
Complete secondary	198	35 (17.7)	1.13 (0.62–2.06)		
Tertiary: university/college/vocational	51	15 (29.4)	1.84 (0.84–4.07)		
Religion			P<0.001	P = 0.034	
Christian	917	163 (17.8)	1	1	
Muslim	142	2 (1.4)	15.02 (3.68–61.28)	0.02 (0.00–0.46)	0.014
Other	22	6 (27.3)	26.25 (4.88–141.1)	1.45 (0.53–3.95)	0.425
Ethnicity			P = 0.010	P = 0.866	
Somali	90	2 (2.2)	1	1	
Kamba	208	31 (14.9)	7.71 (1.80–32.94)	0.17 (0.01–3.42)	0.247
Kikuyu	319	54 (16.9)	8.97 (2.14–37.53)	0.22 (0.01–4.25)	0.314
Kisii	51	8 (15.7)	8.19 (1.67–40.22)	0.18 (0.01–3.84)	0.269
Luhya	176	35 (19.9)	10.92 (2.56–46.54)	0.23 (0.01–4.56)	0.335
Luo	176	37 (21.0)	11.31 (2.66–48.17)	0.22 (0.01–4.34)	0.319
Other	61	4 (6.6)	3.09 (0.55–17.41)	0.25 (0.01–4.89)	0.360
Ever & recent employment/income generating activity			P = 0.032	P = 0.595	
Never	776	109 (14)	1	1	
Yes, not in last month	172	31 (18)	1.35 (0.87–2.09)	0.83 (0.50–1.38)	0.467
Yes, in last month	133	31 (23.3)	1.78 (1.13–2.81)	1.11 (0.65–1.89)	0.697
Ever had sex			P<0.001	P<0.001	
No	439	65 (10.1)	1	1	
Yes	642	106 (24.3)	2.82 (2.01–3.95)	4.52 (2.68–7.63)	<0.001
Slept hungry at night past 4 weeks			P = 0.005	P = 0.004	
No	729	98 (13.4)	1	1	
Yes	352	73 (20.7)	1.62 (1.16–2.27)	1.73 (1.19–2.53)	0.004
Self-assessed household economic situation			P = 0.710		
Very poor	139	24 (17.3)	1		
Moderately poor	858	136 (15.9)	0.89 (0.56–1.44)		
Not poor	84	11 (13.1)	0.72 (0.33–1.56)		
Wealth quantile			P = 0.158		
Poor	361	67 (18.6)	1		
Medium	360	46 (12.8)	0.67 (0.45–1.01)		
Wealthy	360	58 (16.1)	0.84 (0.57–1.24)		
Knows HIV status					
No	133	12 (9.0)	1	1	
Yes	948	158 (16.7)	2.02 (1.09–3.74)	1.20 (0.60–2.37)	

Model 1 is unadjusted logit model; Model 2 is invited to DREAMS, site, and age adjusted logit model for each predictor; Model 3 is fully adjusted multivariable logit model. Variables significant at P<0.10 in model 2 were included in model 3. Invited to DREAMS, site, and age were included even if they were not significant as we wished to adjust for their impact. The capital P is likelihood ratio rest (LRT) p-value

### Severity of physical violence among 15–22 year-olds

Of 1081 AGYW aged 15–22 years, 9.2% (95%CI 7.57–11.03%) experienced moderate physical violence, and 13.8% (95%CI 11.9–15.97%) experienced severe physical violence in the past year. S4 Table shows these proportions for several demographic characteristics. In general, severe physical violence was more prevalent than moderate physical violence levels. Across the levels of the demographic characteristics, moderate violence was higher among AGYW not invited to participate in DREAMS than those invited, resided in Korogocho compared to Viwandani, 18–22 year-olds than 15–17 year-olds, previously or currently married/living with a partner compared to those who had never married or lived with a partner, not in school, ever had sex, slept hungry, and were poor. Severe physical violence was higher among Korogocho residents compared to Viwandani residents, previously married/living with partner, not currently in school, with some secondary education or lower, Christians and other religious groups than Muslims, among Luos and Luhyas, ever had sex, slept hungry at night last 4 weeks, assessed their household economic situation as not poor, or were in the poor wealth quantile.

### Predictors of violence among 15–22 year-olds

Tables 2–4 show the logistic regression model results for psychological, physical, and sexual violence, respectively. From Table 2, AGYW who were previously married/lived with partner, or engaged in employment/income generating activity last month, or slept hungry at night during past 4 weeks had greater odds of experiencing psychological violence. Table 3 shows the odds of experiencing physical violence were lower among those who lived in Viwandani slum relative to those living in Korogocho, and among Muslims; and the odds were greater among AGYW who were previously married or lived with a partner, or slept hungry at night during the past 4 weeks. It can be seen from Table 4 that the odds of sexual violence were lower among AGYW aged 18–22 years (compared to the 15–17 year-olds) and among Muslims (compared to Christians). Sexual violence was higher among AGYW who reported to have ever had sex, or slept hungry at night during the past 4 weeks.

In summary, sleeping hungry at night during past 4 weeks was found to be significantly associated with greater odds for all three forms of violence, other factors held constant. Being a Muslim was associated with lower levels of physical and sexual violence.

## Discussion

This study provides data on the prevalence, levels of and determinants for violence against AGYW in slums settings of Nairobi. This is in line with and a response to a call by the WHO a decade ago urging researchers to incorporate violence against women components into HIV and AIDS prevention and adolescent health promotion programs [21]. Violence against AGYW in Korogocho and Viwandani slums is common, with about four in ten AGYW aged 10–14 years and 15–22 years reporting to have experienced violence in the past six and 12 months, respectively. In both age categories psychological violence was the most experienced, followed by physical violence, and sexual violence. These levels of violence are in line with previous studies that have shown that AGYW in Kenya are predisposed to physical, psychological and sexual violence [2, 33]. In a study by Mathur et al (2018), about two in ten AGYW aged 15–24 reported sexual violence by an intimate partner in the 12 months preceding the study. More concerning, however, is our finding that at 10–14 years, about six in ten of the young girls had ever experienced violence.

Our finding that having ever slept hungry in the last one month increased the odds of experiencing physical, psychological or sexual violence is similar to findings from other studies conducted in sub-Saharan Africa (SSA) and elsewhere. A study conducted in Botswana found that women who were food insecure were more likely to experience sexual violence [34]. Food insufficiency has been linked to high-risk sexual behavior and sexual vulnerability among women [35, 36]. The high levels of food insecurity reported within the NUHDSS [23] are therefore likely to contribute to the high likelihood of AGYW experiencing physical, sexual and psychological violence. Food insecurity puts women at greater risk of violence through: 1) path of stress by causing hunger and worry about having sufficient access to food, which might act as a trigger to interpersonal violence; 2) making it difficult to walk out of an abusive relationship due to dependence for food; 3) engaging in transactional sex as a means of getting food; and so on [37].

Marital status was associated with experiences of physical and psychological violence. Whereas being in a current marital union reduced the likelihood of experiencing psychological violence, having been in a marital union previously increased the chances of experiencing both physical and psychological violence. The finding here that the odds of psychological abuse among formerly married women were more than twice that for single women may point to abuse as a reason for termination of such relationships. The 2014 Kenya Demographic and Health Survey shows that a greater proportion of women and men who were divorced, separated or widowed reported to have ever experienced physical, sexual and emotional violence compared with those who were in marital unions [38]. Other studies have also shown that the risk of violence increases considerably when women want to leave, are trying to leave, are in the process of leaving, or have left a relationship [39–42].

There seem to be some protective norms/cultures. Religion was found to be significantly associated with experiencing violence. Overall, AGYW who identified themselves as Muslims had lower likelihood of experiencing all three forms of violence. This finding is consistent with past research where being religious has been associated with lower likelihood of perpetrating violence [43]. The modalities of most religions encourage peaceful co-existence at individual, family and community levels and also are likely to provide support services for their congregants to resolve conflicts. However, religion may also increase the vulnerability of AGYW as it discourages dissolution of marriages and thus it may encourage a victim to stay in an abusive marriage/relationship. Nevertheless, it is not clear whether they are indeed protected or it is an issue of reporting bias. A previous study found tolerant attitudes of Muslim women towards violence, which is a portrayal of the religious restrictions they have to abide by [44], and which could contribute to under reporting.

AGYW who were in employment in the past one month were more likely to experience psychological violence. The autonomy and independence that often comes with financial freedom has been identifiable as risk for abuse in other studies (UNICEF, 2007). This finding reflects social/cultural attitudes towards women’s employment and may be closely associated with the belief that men hold power in household allocation of resources and decision-making on household expenditures [45]. This may imply that women who work may have little or no decision-making powers on how their income is utilized. Future studies focusing on this can provide valuable data. Findings from other studies in SSA [46, 47] suggest that women form a higher proportion of people working in the informal sector, and that they experience exploitation, work for long hours, are underpaid and are engaged in other forms of work beyond their contractual agreement, factors that are likely to contribute to psychological violence.

Korogocho slum is characterized by higher levels of poverty, low education attainment and violent crime. These directly or indirectly have implications on the risk of physical violence amongst AGYW which, in Viwandani, is estimated to be lower by almost 50%.

The fact that the propensity of experiencing sexual violence among older AGYW was lower by almost 40%, may point to evidence from previous studies that has shown that while the perpetuators are often thought to be strangers, a lot of this is by close family members or friends who take advantage of young AGYW who are not empowered to resist or report such advances.

Our finding that having had sexual intercourse increased the odds of experiencing sexual violence was expected and is in line with previous literature [48]. Sexual experience in this study includes sexual intercourse that AGYW were forced to participate in, including rape, and this could explain the increased odds of sexual violence among those who had had sex. However, we did not distinguish between intimate partner and non-partner sexual violence.

We found no significant association between DREAMS invitation and any domain of violence using these first round data. This could be attributed to the fact that the first round of data collection took place when DREAMS intervention had just started. Given the staggered roll-out of interventions, we do not expect DREAMS to have had prevented violence at this early stage of the program. Second, we have no ‘baseline’ to measure change in violence over the course of that year.

In summary, our study has found high prevalence of and have identified some protective and risk factors for violence among AGYW in two Nairobi slums. The high prevalence among the younger age group (10–14 years), in particular, calls for urgent intervention as childhood exposure to violence has been reported to be a risk factor for violence in adulthood. Our findings can help the formulation and implementation of both national and sub-national policies, budgets and actions to reduce, eliminate and mitigate the consequences of violence against women. As data used in this paper are part of an independent Impact Evaluation, the learnings from these results have not been used to influence DREAMS implementation. However, our finding of no difference in violence among those invited to the program and those not invited among AGYW aged 15–22 years raises a flag for us to pay attention to measurement details which, if okay, might point to the fact that since social change tends to take long to happen, the implementation period might need to be longer. We also note that violence seems to occur together with other forms of social vulnerability such as food insecurity, which is very important in situations where single interventions might fail to lead to expected outcomes. Within DREAMS [21], promising interventions such as gender norms training, school-based HIV and violence prevention programs, and lessons and tools from SASA! intervention in Uganda [49], exist that need to be scaled-up. In rural South Africa, the Intervention with Microfinance for AIDS and Gender Equity (IMAGE) combined a microfinance program with a gender and HIV education to reduce risk of gender based violence and HIV [15]. Other promising strategies to leverage also exist [50].

This study has some limitations. Whereas all due care was taken to ensure that the tools captured the right information, we cannot rule out the possibility of misreporting. It has been shown in other studies that to avoid feeling embarrassed, women tend to under-report physical and sexual violence, such as rape, as is often the case might be blamed on the victim [51–53]. The slum population is generally unique with different social challenges compared to their rural and urban non-slum sub-populations. Therefore, results drawn from this study may not accurately be generalizable to the entire population of AGYW in Kenya. Whenever data allow, there is need to make comparisons across sub-populations and over time to be able to fully understand the dynamics and general trend of occurrence of violence and possibly link it to other health and social outcomes such HIV acquisition, pregnancy outcomes and schooling outcomes among others. Another limitation is that there is no unified framework for classifying different violence domains into moderate or severe. In the present study, we have classified physical violence only into “moderate” or “severe” following WHO guidelines. Standard guidelines for such classifications need to be developed for the other violence domains as well to allow for studying on severity of violence in settings such as ours. Finally, whereas this study has a unique sample given the age of the cohorts and the setting, the use of two different tools could not allow us to immediately compare the two age groups.

## Conclusions

Physical, psychological and sexual violence among AGYW in the two Nairobi slums is common. The violence is intimately related to some of the social as well as cultural norms but, importantly, seem to be driven by the economic circumstances under which these girls find themselves in. Given the association between violence and HIV acquisition in young women, addressing violence against women and girls is critical to curbing the HIV epidemic overall and interventions against this should be supported and promoted.

## Supporting information

S1 Text Computational details and model fit assessment. (DOCX)

S1 Table Collinearity diagnostics with and without the variables ‘ever been pregnant’ and ‘ever given birth’. (DOCX)

S2 Table Prevalence of violence among girls aged 10–14 years. (DOCX)

S3 Table Factors associated with experience of violence among girls aged 10–14yrsꝉ. (DOCX)

S4 Table AGYW ages 15–22 years who experienced moderate and severe violence past 12 months by demographic characteristics. (DOCX)
