# Supplementary material for: Experiences of violence among adolescent girls and young women in Nairobi’s informal settlements prior to scale-up of the DREAMS Partnership: Prevalence, severity and predictors
Source: PLoS One. 2020 Apr 22;15(4):e0231737. doi: 10.1371/journal.pone.0231737 (PMC7176122; doi:10.1371/journal.pone.0231737)
Supplement: S1 Text — (DOCX) [file pone.0231737.s001.docx]

**Computational details and model fit assessment**

Data management and all analyses were performed using STATA v14 (StataCorp, College Station, TX). We fitted a model of the following form, for the binary outcomes (i.e., abused=1 or not abused=0):

$$P\left( \boldsymbol{y}_{\boldsymbol{i}}\boldsymbol{=}1 \right)=\alpha+\boldsymbol{\beta}^{\boldsymbol{'}}\boldsymbol{x}_{i}\boldsymbol{,}i=1,2,3, \ldots, n$$

such that $n$ is the number of AGYW, $\boldsymbol{y}_{\boldsymbol{i}}$is the response vector for girl/young woman $i$, $\alpha$ is the intercept, $\boldsymbol{x}$is the vector of explanatory variables described in the methods section of the main text, and $\boldsymbol{\beta}$ is the matrix of unknown coefficients associated with the explanatory variables.

For the ordinal physical violence outcome, the model can be expressed as:

$$P\left( \boldsymbol{y}_{\boldsymbol{i}}\boldsymbol{\leq}j \right)=\alpha_{j}+\boldsymbol{\beta}^{\boldsymbol{'}}\boldsymbol{x}_{i}\boldsymbol{,}i=1,2, \ldots, n; j=1,\ldots, c-1$$

where $\alpha_{s}$ are now the thresholds and $c$ is the number of levels of the ordinal outcome, $\boldsymbol{y}$. In our case, $c=3$. The other parameters have the same meanings as before. For each of the three violence outcomes, we evaluated three plausible link functions, that is, logit, probit, and complementary log-log (clog-log).

To check the predictive power of the models, we proceeded as follows. For the dichotomous outcomes (i.e., psychological and sexual violence), we estimated the area under the Receiver Operating Characteristic curve (denoted AUC) ([Hosmer Jr, Lemeshow, & Sturdivant, 2013](#_ENREF_2)). For the ordinal physical violence, we estimated the concordance statistic ([Harrell, Lee, & Mark, 1996](#_ENREF_1)). We use C-statistic to refer to both measures. The C-statistic is a measure of predictive power of a model that strictly uses ordinality and is natural for models that imply stochastic orderings at various settings of the explanatory variable(s). It ranges from 0.5 (i.e., the null value) to 1. The higher the value above 0.5, the better the predictive power of the model.

**Summary of model fit evaluations**

Whereas the three link functions performed approximately equally well based on the log likelihood value, the logit model generally provided a better fit (psychological= - 646.27; physical= -713.80; sexual= -425.72) than the probit model (psychological= -646.40; physical= -713.28; sexual= -426.81) and the clog-log model (psychological= -646.47; physical= -714.64; sexual= -424.96). The logit model was preferred as it also has the advantage of the ability to obtain odds ratios which are more appealing and intuitive in quantifying associations. Table S3 shows the C-statistic using multivariable logistic regression model. The C-statistics were all significant and indicated, in general, acceptable model fit [C-statistics: Psychological violence= 0.66 (95%CI: 0.62-0.69); Physical violence= 0.66 (95%CI: 0.61-0.70); and Sexual violence= 0.71 (95%CI: 0.67-0.75)]. As an example, for sexual violence, the 0.71 (95%CI: 0.67-0.75) C-statistic value implies that for 70% (95%CI: 63-77%) of the untied pairs on sexual violence, the observation with the higher sexual violence score also had a stochastically higher estimated distribution.

**References**

Harrell, F. E., Lee, K. L., & Mark, D. B. (1996). Multivariable prognostic models: issues in developing models, evaluating assumptions and adequacy, and measuring and reducing errors. *Statistics in medicine, 15*(4), 361-387.

Hosmer Jr, D. W., Lemeshow, S., & Sturdivant, R. X. (2013). *Applied logistic regression* (Vol. 398): John Wiley & Sons.
