# Supplementary material for: Experiences of violence among adolescent girls and young women in Nairobi’s informal settlements prior to scale-up of the DREAMS Partnership: Prevalence, severity and predictors
Source: PLoS One. 2020 Apr 22;15(4):e0231737. doi: 10.1371/journal.pone.0231737 (PMC7176122; doi:10.1371/journal.pone.0231737)
Supplement: S1 Table — (DOCX) [file pone.0231737.s002.docx]

**S1 Table. Collinearity diagnostics with and without the variables ‘ever been pregnant’ and ‘ever given birth’**

| **Variable** | **VIF** | **SQRT VIF** | **Tolerance** | **R-Squared** |
| --- | --- | --- | --- | --- |
| Invited to DREAMS | 1.1 | 1.060 | 0.898 | 0.102 |
| Site | 1.7 | 1.320 | 0.578 | 0.422 |
| Age | 1.4 | 1.200 | 0.695 | 0.305 |
| Marital status | 2.0 | 1.400 | 0.513 | 0.487 |
| Currently in school | 1.4 | 1.200 | 0.692 | 0.308 |
| Education level | 1.3 | 1.160 | 0.745 | 0.255 |
| Religion | 1.3 | 1.120 | 0.798 | 0.202 |
| Ethnicity | 1.2 | 1.100 | 0.820 | 0.180 |
| Employment/income generating activity | 1.2 | 1.090 | 0.837 | 0.163 |
| Ever had sex | 371.1 | 19.260 | 0.003 | 0.997 |
| Ever been pregnant | 1061.9 | 32.590 | 0.001 | 0.999 |
| Ever given birth | 883.9 | 29.730 | 0.001 | 0.999 |
| Slept hungry at night in past 4 weeks | 1.3 | 1.120 | 0.794 | 0.206 |
| Self-assessed poverty level | 1.1 | 1.060 | 0.896 | 0.104 |
| Wealth quantile | 1.8 | 1.330 | 0.567 | 0.433 |
| **Variable** | **VIF** | **SQRT VIF** | **Tolerance** | **R-Squared** |
| Invited to DREAMS | 1.1 | 1.04 | 0.917 | 0.083 |
| Site | 1.6 | 1.27 | 0.623 | 0.377 |
| Age | 1.9 | 1.39 | 0.519 | 0.481 |
| Marital status | 1.7 | 1.32 | 0.577 | 0.423 |
| Currently in school | 1.9 | 1.37 | 0.534 | 0.466 |
| Education level | 1.3 | 1.14 | 0.767 | 0.233 |
| Religion | 1.3 | 1.13 | 0.787 | 0.213 |
| Ethnicity | 1.2 | 1.09 | 0.839 | 0.161 |
| Employment/income generating activity | 1.4 | 1.17 | 0.728 | 0.272 |
| Ever had sex | 1.0 | 1.00 | 0.993 | 0.007 |
| Slept hungry at night in past 4 weeks | 1.0 | 1.01 | 0.983 | 0.017 |
| Self-assessed poverty level | 1.0 | 1.02 | 0.959 | 0.041 |
| Wealth quantile | 1.6 | 1.26 | 0.630 | 0.371 |
