# Supplementary material for: Experiences of violence among adolescent girls and young women in Nairobi’s informal settlements prior to scale-up of the DREAMS Partnership: Prevalence, severity and predictors
Source: PLoS One. 2020 Apr 22;15(4):e0231737. doi: 10.1371/journal.pone.0231737 (PMC7176122; doi:10.1371/journal.pone.0231737)
Supplement: S2 Table — (DOCX) [file pone.0231737.s003.docx]

**S2 Table.** Prevalence of violence among girls aged 10-14 years

|  | Total | Past 6 months | |  | Ever experienced | |
| --- | --- | --- | --- | --- | --- | --- |
| Characteristics | N | n | % |  | n | % |
| Overall | 606 | 229 | 37.8 |  | 348 | 57.4 |
|  |  |  |  |  |  |  |
| Invitation to DREAMS |  |  |  |  |  |  |
| Not invited | 316 | 123 | 38.9 |  | 197 | 62.3 |
| Invited | 290 | 106 | 36.6 |  | 151 | 52.1 |
| DSS study site |  |  |  |  |  |  |
| Korogocho | 323 | 125 | 38.7 |  | 174 | 53.9 |
| Viwandani | 283 | 104 | 36.7 |  | 174 | 61.5 |
| Age groups |  |  |  |  |  |  |
| 10-12yr | 372 | 147 | 39.5 |  | 222 | 59.7 |
| 13-14yr | 234 | 82 | 35.0 |  | 126 | 53.8 |
| Currently enrolled in school? |  |  |  |  |  |  |
| No | 5 | 2 | 40.0 |  | 3 | 60.0 |
| Yes | 601 | 227 | 37.8 |  | 345 | 57.4 |
| School grade |  |  |  |  |  |  |
| Upper primary or Secondary | 257 | 87 | 33.9 |  | 138 | 53.7 |
| Middle primary | 311 | 127 | 40.8 |  | 190 | 61.1 |
| Lower primary | 38 | 15 | 39.5 |  | 20 | 52.6 |
| School type |  |  |  |  |  |  |
| Public school | 271 | 108 | 39.9 |  | 164 | 60.5 |
| Private, non-religious or secular school | 260 | 88 | 33.8 |  | 138 | 53.1 |
| Religious school | 70 | 31 | 44.3 |  | 43 | 61.4 |
| Not enrolled in school | 5 | 3 | 60.0 |  | 2 | 40.0 |
| Gender of teachers |  |  |  |  |  |  |
| Mostly women (very few or no men) | 162 | 52 | 32.1 |  | 84 | 51.9 |
| Mostly men (very few or no women) | 58 | 22 | 37.9 |  | 32 | 55.2 |
| Both men and women | 381 | 153 | 40.2 |  | 229 | 60.1 |
| Not enrolled | 5 | 3 | 60.0 |  | 2 | 40.0 |
| Religion |  |  |  |  |  |  |
| Christian | 534 | 207 | 38.8 |  | 316 | 59.2 |
| Muslim | 61 | 18 | 29.5 |  | 26 | 42.6 |
| Other | 11 | 4 | 36.4 |  | 6 | 54.5 |
| Ethnic group |  |  |  |  |  |  |
| Somali | 56 | 17 | 30.4 |  | 24 | 42.9 |
| Kamba | 96 | 37 | 38.5 |  | 55 | 57.3 |
| Kikuyu | 195 | 71 | 36.4 |  | 105 | 53.8 |
| Kisii | 34 | 12 | 35.3 |  | 25 | 73.5 |
| Luhya | 91 | 38 | 41.8 |  | 60 | 65.9 |
| Luo | 115 | 48 | 41.7 |  | 68 | 59.1 |
| Other | 19 | 6 | 31.6 |  | 11 | 57.9 |
| Activities for payment past 6 months |  |  |  |  |  |  |
| no | 577 | 212 | 36.7 |  | 325 | 56.3 |
| yes | 29 | 17 | 58.6 |  | 23 | 79.3 |
| Ever had sex |  |  |  |  |  |  |
| no | 594 | 220 | 37.0 |  | 336 | 56.6 |
| yes | 12 | 9 | 75.0 |  | 12 | 100.0 |
| Family did not have enough food due to money | |  |  |  |  |  |
| no | 228 | 75 | 32.9 |  | 117 | 51.3 |
| yes | 378 | 154 | 40.7 |  | 231 | 61.1 |
