# Supplementary material for: Experiences of violence among adolescent girls and young women in Nairobi’s informal settlements prior to scale-up of the DREAMS Partnership: Prevalence, severity and predictors
Source: PLoS One. 2020 Apr 22;15(4):e0231737. doi: 10.1371/journal.pone.0231737 (PMC7176122; doi:10.1371/journal.pone.0231737)
Supplement: S3 Table — (DOCX) [file pone.0231737.s004.docx]

**S3 Table. Factors associated with experience of violence among girls aged 10-14yrsꝉ**

|  | Total | Ever experienced any act of violence n (%) | Model 1 | Model 2 | Model 3 | |
| --- | --- | --- | --- | --- | --- | --- |
| Characteristics | N |  | Unadjusted OR (95%CI) | Invited, site, and age adjusted OR (95%CI) | Fully adjusted OR (95%CI) | p-value |
| Overall | 606 | 229 (37.8) |  |  |  |  |
|  |  |  |  |  |  |  |
| Invitation to DREAMS |  |  | P=0.011 | P=0.044 | P=0.026 |  |
| Not invited | 316 | 123 (38.9) | 1 | 1 | 1 |  |
| Invited | 290 | 106 (36.6) | 0.66 (0.47-0.91) | 0.71 (0.51-0.99) | 0.68 (0.48-0.95) | 0.026 |
| DSS study site |  |  | P=0.059 | P=0.175 | P=0.087 |  |
| Korogocho | 323 | 125 (38.7) | 1 | 1 | 1 |  |
| Viwandani | 283 | 104 (36.7) | 1.37 (0.99-1.89) | 1.26 (0.9-1.77) | 1.37 (0.96-1.96) | 0.087 |
| Age groups |  |  | P=0.158 | P=0.222 | P=0.126 |  |
| 10-12yr | 372 | 147 (39.5) | 1 | 1 | 1 |  |
| 13-14yr | 234 | 82 (35.0) | 0.79 (0.57-1.1) | 0.81 (0.58-1.13) | 0.77 (0.54-1.08) | 0.126 |
| Currently enrolled in school? |  |  | P=0.907 | P=0.903 |  |  |
| No | 5 | 2 (40.0) | 1 | 1 |  |  |
| Yes | 601 | 227 (37.8) | 0.9 (0.15-5.42) | 0.89 (0.15-5.48) |  |  |
| School grade |  |  | P=0.172 | P=0.242 |  |  |
| Upper primary or Secondary | 257 | 87 (33.9) | 1 | 1 |  |  |
| Middle primary | 311 | 127 (40.8) | 1.35 (0.97-1.89) | 1.39 (0.91-2.12) |  |  |
| Lower primary | 38 | 15 (39.5) | 0.96 (0.48-1.9) | 0.99 (0.47-2.11) |  |  |
| School type |  |  | P=0.172 | P=0.165 |  |  |
| Public school | 271 | 108 (39.9) | 1 | 1 |  |  |
| Private, non-religious or secular school | 260 | 88 (33.8) | 0.74 (0.52-1.04) | 0.77 (0.54-1.09) |  |  |
| Religious school | 70 | 31 (44.3) | 1.04 (0.61-1.78) | 1.20 (0.68-2.11) |  |  |
| Not enrolled in school | 5 | 3 (60.0) |  |  |  |  |
| Gender of teachers |  |  | P=0.193 | P=0.325 |  |  |
| Mostly women (very few or no men) | 162 | 52 (32.1) | 1 | 1 |  |  |
| Mostly men (very few or no women) | 58 | 22 (37.9) | 1.14 (0.63-2.09) | 1.21 (0.66-2.22) |  |  |
| Both men and women | 381 | 153 (40.2) | 1.40 (0.97-2.03) | 1.33 (0.92-1.95) |  |  |
| Not enrolled in school | 5 | 3 (60) |  |  |  |  |
| Religion |  |  | P=0.049 | P=0.074 | P=0.120 |  |
| Christian | 534 | 207 (38.8) | 1 | 1 | 1 |  |
| Muslim | 61 | 18 (29.5) | 0.51 (0.3-0.88) | 0.54 (0.31-0.93) | 0.57 (0.32-0.99) | 0.045 |
| Other | 11 | 4 (36.4) | 0.83 (0.25-2.75) | 0.72 (0.21-2.42) | 0.72 (0.2-2.51) | 0.602 |
| Ethnic group |  |  | P=0.0621 | P=0.145 |  |  |
| Somali | 56 | 17 (30.4) | 1 | 1 |  |  |
| Kamba | 96 | 37 (38.5) | 1.79 (0.92-3.48) | 1.64 (0.81-3.32) |  |  |
| Kikuyu | 195 | 71 (36.4) | 1.79 (0.92-3.48) | 1.64 (0.81-3.32) |  |  |
| Kisii | 34 | 12 (35.3) | 1.56 (0.85-2.83) | 1.53 (0.83-2.83) |  |  |
| Luhya | 91 | 38 (41.8) | 3.7 (1.46-9.37) | 2.92 (1.1-7.76) |  |  |
| Luo | 115 | 48 (41.7) | 2.58 (1.3-5.12) | 2.49 (1.24-4.98) |  |  |
| Other | 19 | 6 (31.6) | 1.93 (1.01-3.68) | 1.95 (1.02-3.75) |  |  |
| Activities for payment past 6 months |  |  | P=0.019 | P=0.013 | P=0.017 |  |
| no | 577 | 212 (36.7) | 1 | 1 | 1 |  |
| yes | 29 | 17 (58.6) | 2.97 (1.19-7.41) | 3.19 (1.27-7.99) | 3.09 (1.23-7.77) | 0.017 |
| Ever had sex |  |  |  |  |  |  |
| no | 594 | 220 (37.0) | 1 |  |  |  |
| yes | 12 | 9 (75.0) | *__* |  |  |  |
| Family did not have enough food due to money | |  | P=0.018 | P=0.002 | P=0.003 |  |
| no | 228 | 75 (32.9) | 1 | 1 | 1 |  |
| yes | 378 | 154 (40.7) | 1.49 (1.07-2.08) | 1.73 (1.22-2.46) | 1.69 (1.19-2.41) | 0.003 |

*__* The model could not run for ‘ever had sex’ explanatory variable because of separation problem. ^ꝉ^The binary outcome here is whether the girl had ever experienced any act of violence. Model 1 is unadjusted logit model; Model 2 is invited to DREAMS, site, and age adjusted logit model for each predictor; Model 3 is fully adjusted multivariable logit model. Variables significant at P<0.10 in model 2 were included in model 3. Invited to DREAMS, site, and age were included even if they were not significant as we wished to adjust for their impact. The capital P is likelihood ratio rest (LRT) p-value
