# Supplementary material for: Experiences of violence among adolescent girls and young women in Nairobi’s informal settlements prior to scale-up of the DREAMS Partnership: Prevalence, severity and predictors
Source: PLoS One. 2020 Apr 22;15(4):e0231737. doi: 10.1371/journal.pone.0231737 (PMC7176122; doi:10.1371/journal.pone.0231737)
Supplement: S4 Table — (DOCX) [file pone.0231737.s005.docx]

**S4 Table.** AGYW ages 15-22 years who experienced moderate and severe violence past 12 months by demographic characteristics

| Characteristics | Level of violence experience last 12 months | | | | | |
| --- | --- | --- | --- | --- | --- | --- |
|  | Total | Moderate | |  | Severe | |
|  | N | n | % |  | n | % |
| Overall (all AGYW 15-22yrs) | 1,081 | 99 | 9.2 |  | 149 | 13.8 |
|  |  |  |  |  |  |  |
| Invited to DREAMS |  |  |  |  |  |  |
| Not-invited | 545 | 64 | 10.4 |  | 71 | 13.0 |
| Invited | 536 | 35 | 7.5 |  | 78 | 14.6 |
| Site/slum |  |  |  |  |  |  |
| Korogocho | 617 | 64 | 10.4 |  | 108 | 17.5 |
| Viwandani | 464 | 35 | 7.5 |  | 41 | 8.8 |
| Age |  |  |  |  |  |  |
| 15-17yrs | 547 | 40 | 7.3 |  | 79 | 14.4 |
| 18-22yrs | 534 | 59 | 11.0 |  | 70 | 13.1 |
| Marital/co-habitation status |  |  |  |  |  |  |
| Never married | 843 | 68 | 8.1 |  | 110 | 13.0 |
| Previously married/living with partner | 33 | 4 | 12.1 |  | 11 | 33.3 |
| Currently married/living with partner | 205 | 27 | 13.2 |  | 28 | 13.7 |
| Currently in school |  |  |  |  |  |  |
| No | 455 | 48 | 10.5 |  | 74 | 16.3 |
| Yes | 626 | 51 | 8.1 |  | 75 | 12.0 |
| Educational level |  |  |  |  |  |  |
| None/some primary | 124 | 13 | 10.4 |  | 23 | 18.4 |
| Complete primary | 217 | 18 | 8.3 |  | 38 | 17.5 |
| Some secondary | 491 | 41 | 8.4 |  | 67 | 13.6 |
| Complete secondary | 198 | 24 | 12.1 |  | 17 | 8.6 |
| Tertiary: university/college/vocational | 51 | 3 | 6.0 |  | 4 | 8.0 |
| Religion |  |  |  |  |  |  |
| Muslim | 142 | 8 | 5.6 |  | 11 | 7.7 |
| Christian | 917 | 89 | 9.7 |  | 134 | 14.6 |
| Other | 22 | 2 | 9.1 |  | 4 | 18.2 |
| Ethnicity |  |  |  |  |  |  |
| Somali | 90 | 6 | 6.7 |  | 6 | 6.7 |
| Kamba | 208 | 20 | 9.6 |  | 20 | 9.6 |
| Kikuyu | 319 | 31 | 9.7 |  | 42 | 13.2 |
| Kisii | 51 | 6 | 11.8 |  | 6 | 11.8 |
| Luhya | 176 | 16 | 9.1 |  | 27 | 15.3 |
| Luo | 176 | 17 | 9.7 |  | 40 | 22.7 |
| Other | 61 | 3 | 4.9 |  | 8 | 13.1 |
| Ever & recent employment/income generating activity | | |  |  |  |  |
| Never | 776 | 63 | 8.1 |  | 103 | 13.3 |
| Yes, not in last month | 172 | 19 | 11.0 |  | 24 | 14.0 |
| Yes, in last month | 133 | 17 | 12.8 |  | 22 | 16.5 |
| Ever had sex |  |  |  |  |  |  |
| No | 437 | 48 | 7.5 |  | 76 | 11.8 |
| Yes | 642 | 51 | 11.7 |  | 73 | 16.7 |
| Refused/Unknown | 2 | 0 | 0.0 |  | 0 | 0.0 |
| Slept hungry at night past 4 weeks |  |  |  |  |  |  |
| No | 715 | 62 | 8.6 |  | 83 | 11.6 |
| Yes | 352 | 37 | 10.6 |  | 65 | 18.6 |
| don't know | 14 | 0 | 0.0 |  | 1 | 7.1 |
| Self-assessed household economic situation | |  |  |  |  |  |
| Very poor | 139 | 9 | 6.5 |  | 17 | 12.2 |
| Moderately poor | 858 | 82 | 9.6 |  | 117 | 13.6 |
| Not poor | 84 | 8 | 9.5 |  | 15 | 17.9 |
| Wealth quantile |  |  |  |  |  |  |
| Poor | 361 | 44 | 12.2 |  | 65 | 18.0 |
| Medium | 360 | 25 | 6.9 |  | 45 | 12.5 |
| Wealthy | 360 | 30 | 8.3 |  | 39 | 10.8 |
